# Evaluation of the association of serum glypican-4 with prevalent and future kidney function

**DOI:** 10.1038/s41598-022-14306-7

**Published:** 2022-06-17

**Authors:** Axel Muendlein, Eva Maria Brandtner, Andreas Leiherer, Kathrin Geiger, Christine Heinzle, Stella Gaenger, Peter Fraunberger, Dominik Haider, Christoph H. Saely, Heinz Drexel

**Affiliations:** 1 Vorarlberg Institute for Vascular Investigation and Treatment (VIVIT), Carinagasse 47, 6800 Feldkirch, Austria; 2 Medical Central Laboratories, Feldkirch, Austria; 3 Private University of the Principality of Liechtenstein, Triesen, Liechtenstein; 4 Department of Medicine, Academic Teaching Hospital Bregenz, Bregenz, Austria; 5 Drexel University College of Medicine, Philadelphia, PA USA

**Keywords:** Kidney diseases, Diagnostic markers, Prognostic markers, Translational research

## Abstract

Serum glypican-4 (GPC4) has been identified as an insulin-sensitizing adipokine serving as a marker for body mass index and insulin resistance in humans. The association of circulating GPC4 with kidney function is to date largely unexplored. Therefore, we aimed to evaluate the association between serum GPC4 and prevalent as well future kidney function in a prospective cohort study. The study included 456 Caucasian coronary angiography patients. After a median follow up period of 3.4 years, data on kidney function was reassessed in all patients. Chronic kidney disease (CKD) was defined by decreased estimated glomerular filtration rate (eGFR) < 60 mL/min/1.73 m^2^ or albuminuria. At baseline, serum GPC4 was significantly associated with decreased eGFR (adjusted odds ratio (OR) per standard deviation = 4.75 [2.66–8.48]; *P* <  0.001), albuminuria (OR = 1.49 [1.15–1.92]; *P* = 0.002), and, accordingly, with CKD (OR = 1.75 [1.35–2.26]; *P* < 0.001). GPC4 levels also significantly and independently predicted the incidence of newly diagnosed decreased eGFR (OR = 2.74 [1.82–4.14]; *P* < 0.001, albuminuria (OR = 1.58 [1.01–2.46]; *P* = 0.043, and CKD (OR = 2.16 [1.45–3.23]; *P* < 0.001). ROC analysis indicated an additional predictive value of GPC4 to a basic prediction model for newly diagnosed CKD and eGFR < 60 mL/min/1.73 m^2^. Our study, therefore, indicates that high serum GPC4 is associated with decreased prevalent and future kidney function.

## Introduction

Chronic kidney disease (CKD) is strongly associated with an increased risk of cardiovascular and all-cause mortality^1,2^. Main risk factors for CKD are hypertension and diabetes^3^. Criteria for the definition of CKD are based on decreased glomerular filtration rate (GFR) or markers of kidney damage such as albuminuria^4^. Much interest has focused on further kidney markers to improve early disease management and risk prediction, particularly in high risk subjects such as coronary patients. In this regard, uromodulin^5–7^ and fibroblast growth factor 23 (FGF23)^8–10^ have been proposed as new promising biomarkers of kidney disease.

Adipose tissue has been recognised as a highly active endocrine organ secreting various biologically active adipokines, whose altered expression and secretion have been associated with a number of pathologies, including CKD^11,12^. In 2012, Ussar et al. showed that the cell surface protein glypican-4 (GPC4) also plays the role of an adipokine^13^. GPC4 belongs to the family of heparan sulfate proteoglycans (HSPGs), which are linked to the cell membrane by a glycosylphosphatidylinositol (GPI) anchor and mediate interactions with a variety of extracellular ligands such as growth factors and adhesion molecules^14^. GPC4 as well as other HSPGs can be released from the cell surface into extracellular space by phospholipase‐mediated cleavage of the GPI anchor or by proteolytic shedding^15,16^. Ussar et al. demonstrated that both membrane-bound and released GPC4 can interact with the insulin receptor and enhance insulin signalling in pre-adipocytes promoting adipocyte differentiation^13^. The authors further showed that serum GPC4 is a marker for body mass index (BMI) and insulin resistance in mice and humans^13^, which has been confirmed by subsequent studies^17–20^. Recently, Cha et al. revealed that circulating GPC4 is linked with estimated GFR (eGFR) and urinary albumin excretion in individuals with diabetes^21^. Another study showed a significant correlation of GPC4 with creatinine in patients with metabolic syndrome^22^. However, to our best knowledge, no other reports on the association of serum GPC4 with kidney function exist so far. Moreover, it is unclear, whether GPC4 can predict the development of future decreased kidney function.

In order to get deeper insights into the association of GPC4 with kidney function, we investigated the association of serum GPC4 with CKD as well as with decreased estimated GFR and albuminuria at baseline and after a 3.4-year follow up period in a well characterized cohort of coronary angiography patients. In addition, we evaluated its diagnostic and prognostic value as a new biomarker for decreased kidney function.

## Results

### Baseline

GPC4 levels ranged from 1.6 ng/mL to 12.8 ng/mL; the median GPC4 level was 5.5 [interquartile range: 4.6–6.9] ng/mL. Patients’ baseline characteristics of the total study cohort as well as stratified according to GPC4 quartiles are given in Table 1. Overall, the characteristics of our patients were representative for patients undergoing coronary angiography for the evaluation of coronary artery disease (CAD), with a high prevalence of male gender, type 2 diabetes mellitus (T2DM), and hypertension. Age, BMI, and parameters of kidney function were significantly associated with increasing GPC4 quartiles. Results from correlation analyses confirmed these observations (Table 2). Results from correlation analyses further showed that GPC4 serum levels were significantly linked with systolic blood pressure, urea, uromodulin, and FGF23 (Table 2). Analysis of covariance revealed that serum GPC4 was significantly associated with eGFR and the albumin/creatinine concentration ratio (ACR) independently from classic risk factors for decreased kidney function (each *P-*value < 0.001; Supplemental Table S1). Importantly, serum GPC4 was associated with ACR independently from eGFR (F = 9.3; *P* = 0.002) and with eGFR independently from ACR (F = 113.0; *P* < 0.001).

**Table 1:** Baseline characteristics of the total study cohort as well as stratified according to glypican-4 quartiles.

	Total cohort (n = 456)	Quartile 1 (n = 113)	Quartile 2 (n = 115)	Quartile 3 (n = 114)	Quartile 4 (n = 114)	*P*-value
Glypican-4, ng/mL	5.5 [4.6–6.9]	4.2 [3.9–4.3]	5.0 [4.8–5.3]	6.0 [5.7–6.4]	7.9 [7.3–9.1]	–
Age, years	65 [57–72]	59 [52–66.5]	63 [56–70]	67 [58–73]	70 [62–75]	< 0.001
Male sex, % (n)	62.7 (286)	65.5 (74)	65.2 (75)	61.4 (70)	58.8 (67)	0.238
History of smoking	55.9 (255)	59.3 (67)	60.9 (70)	55.3 (63)	48.2 (55)	0.063
Body mass index, kg/m^2^	27.4 [25.2–30.1]	26.9 [24.9–29.4]	26.5 [24.7–29.4]	27.8 [25.4–30.6]	28.4 [26.1–31.8]	< 0.001
Hypertension, % (n)	79 (360)	78.8 [89]	80.9 [93]	71.9 [82]	84.3 [96]	0.837
T2DM, % (n)^†^	22.9 (104)	18.8 (21)	23.5 (27)	24.6 (28)	24.6 (28)	0.297
Total cholesterol, mg/dL^††^	195 [164–225]	198 [162–235]	197 [174–221]	195 [166–229]	190 [163–221]	0.227
Significant CAD, % (n)	53.1 (242)	56.6 (64)	54.8 (63)	47.4 (54)	53.5 (61)	0.423
eGFR, mL/min/1.73 m^2^	88 [74.9–96.5]	93 [85–101]	91 [84–98]	88 [73–95]	73 [62–85]	< 0.001
eGFR < 60 mL/min/1.73 m^2^, % (n)	7.2 (33)	0.9 (1)	0.0 (0)	7.0 (8)	21.1 (24)	< 0.001
ACR, mg/g	13.0 [7.3–26.4]	10.1 [6.1–21.4]	10.9 [7.2–18.2]	17.6 [9.1–32.7]	16.2 [7.8–38.3]	< 0.001
Albuminuria, % (n)	99 (21.7)	14.2 (16)	13.9 (16)	27.2 (31)	31.6 (36)	< 0.001
Chronic kidney disease, % (n)	25.2 (115)	15.0 [17]	13.9 [16]	30.7 [35]	41.2 [47]	< 0.001
Statin use	52.9 (241)	59.3 (67)	49.6 (57)	53.5 (61)	49.1 (56)	0.207
Beta blocker use	56.8 (259)	61.1 (69)	52.2 (60)	54.4 (62)	59.6 (68)	0.928
ACE inhibitor use	27.4 (125)	25.7 (29)	28.7 (33)	24.6 (28)	30.7 (35)	0.559

^†^Missing samples: n = 1; ^††^missing samples: n = 2. Differences between baseline patients’ characteristics and glypican-4 quartiles were tested for statistical significance with the Chi-squared tests for trend for categorical and Jonckheere Terpstra tests for continuous variables, respectively. Continuous variables are given as median [interquartile range]. T2DM, type 2 diabetes mellitus; HOMA-IR, Homeostatic Model Assessment of Insulin Resistance; CAD, coronary artery disease; eGFR, estimated glomerular filtration rate; ACR, albumin-to-creatinine ratio; ACE, angiotensin converting enzyme.

**Table 2:** Correlation between glypican-4 and baseline clinical and laboratory variables.

	N	Spearman's rho	*P*-value
Age	456	0.314	< 0.001
Body mass index	456	0.180	< 0.001
Systolic blood pressure	454	0.127	0.007
Diastolic blood pressure	454	0.014	0.774
Total cholesterol	456	− 0.068	0.147
HbA1c	456	0.038	0.417
HOMA IR	456	0.058	0.226
C-reactive protein	455	0.056	0.233
eGFR	456	− 0.419	< 0.001
ACR	456	0.187	< 0.001
Creatinine	456	0.252	< 0.001
Urea	456	0.180	< 0.001
Uromodulin	286	− 0.268	< 0.001
FGF23	372	0.256	< 0.001

Association between GPC4 and clinical and laboratory variables were analyzed in accordance with the Spearman rank correlation coefficient analysis. All biomarkers were measured in serum or plasma samples. HbA1c, hemoglobin A1c; HOMA IR, Homeostatic Model Assessment of Insulin Resistance; FGF23, fibroblast growth factor 23.

**Figure 1:**
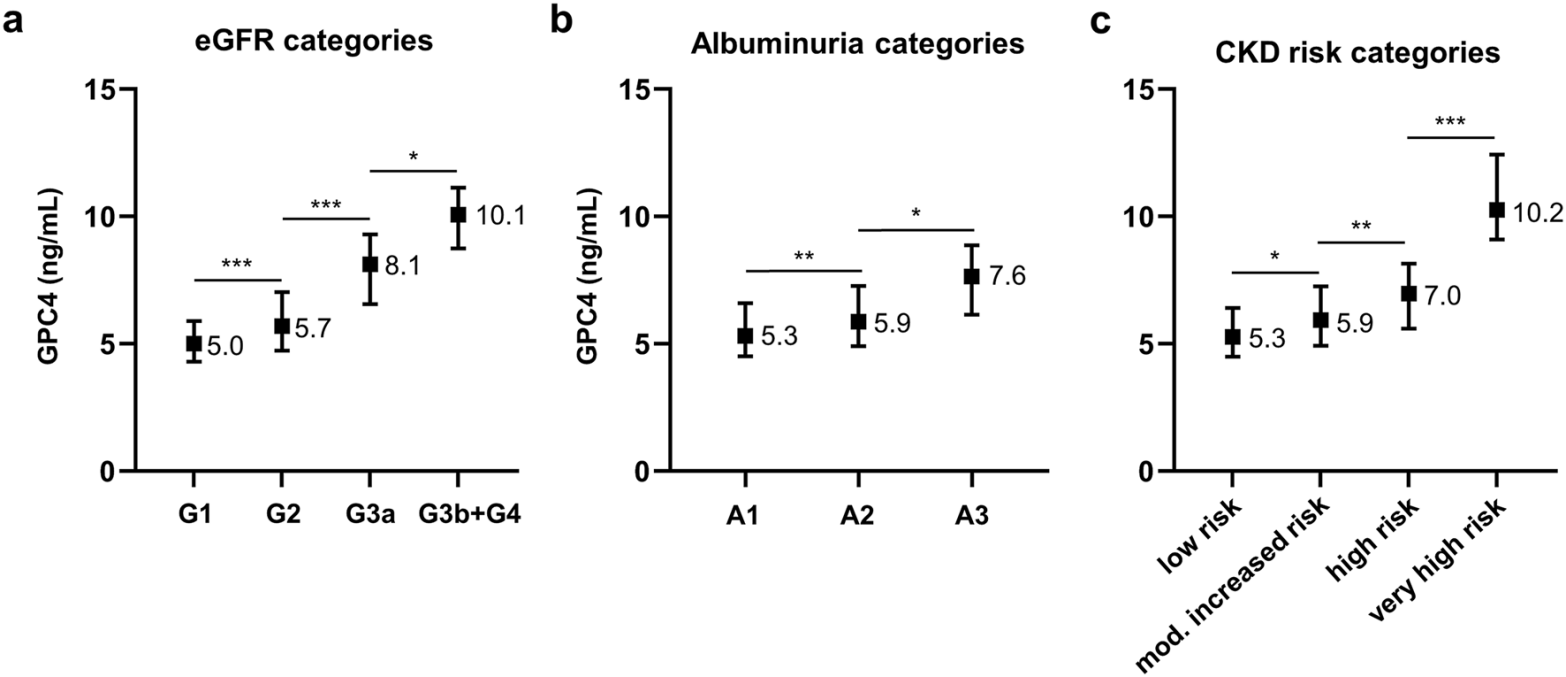
Association between glypican-4 levels and kidney function. Glypican-4 levels are expressed as median with interquartile range. (**a**) Estimated glomerular filtration rate categories are assigned as follows: G1: eGFR ≥ 90 mL/min/1.73 m^2^ (n = 196); G2: eGFR 60–89 mL/min/1.73 m^2^ (n = 227); G3a: eGFR 45–59 mL/min/1.73 m^2^ (n = 23); G3b: eGFR 30–44 mL/min/1.73 m^2^ (n = 9); G4: eGFR 15–29 mL/min/1.73 m^2^ (n = 1); due to the low number of G4 subjects, G4 was combined with G3b; (**b**) Albuminuria categories are based on the urinary albumin-to-creatinine ratio and are assigned as follows: A1: ACR < 30 mg/g (n = 357); A2: 30–300 mg/g (n = 83); A3: ACR > 300 mg/g (n = 16). (**c**) Chronic kidney disease (CKD) risk categories of the prognosis of CKD are based on eGFR and albuminuria categories according to the 2012 KDIGO clinical practice guideline for the evaluation and management of CKD^4^ revealing that 341 subject were at low risk, 84 patients were at moderately increased risk, 20 patients were at high risk, and 11 patients were at very high risk. CKD, chronic kidney disease; GPC4, glypican-4. ***P < 0.001; **P < 0.005; *P < 0.05.

Figure 1a–c shows GPC4 levels stratified by categories of eGFR, albuminuria, and risk of CKD progression, respectively. Increased GPC4 levels were significantly associated with categories of decreased eGFR (Fig. 1a; *P*_trend_ < 0.001). Particularly, serum GPC4 differed highly significantly between patients with normal filtration rates (G1) and mildly decreased filtration rates (G2) and between individuals with mildly decreased filtration rates and moderately decreased filtration rates (G3a). GPC4 differed significantly between categories of severity of albuminuria (Fig. 1b; *P*_trend_ < 0.001) and between risk categories of CKD progression (Fig. 1c; *P*_trend_ < 0.001). Consequently, serum GPC4 was significantly elevated in patients with CKD compared to patients without CKD (median [interquartile range] = 6.4 [5.3–8.0] ng/mL versus 5.3 [4.5–6.4] ng/mL; *P* < 0.001).

**Figure 2:**
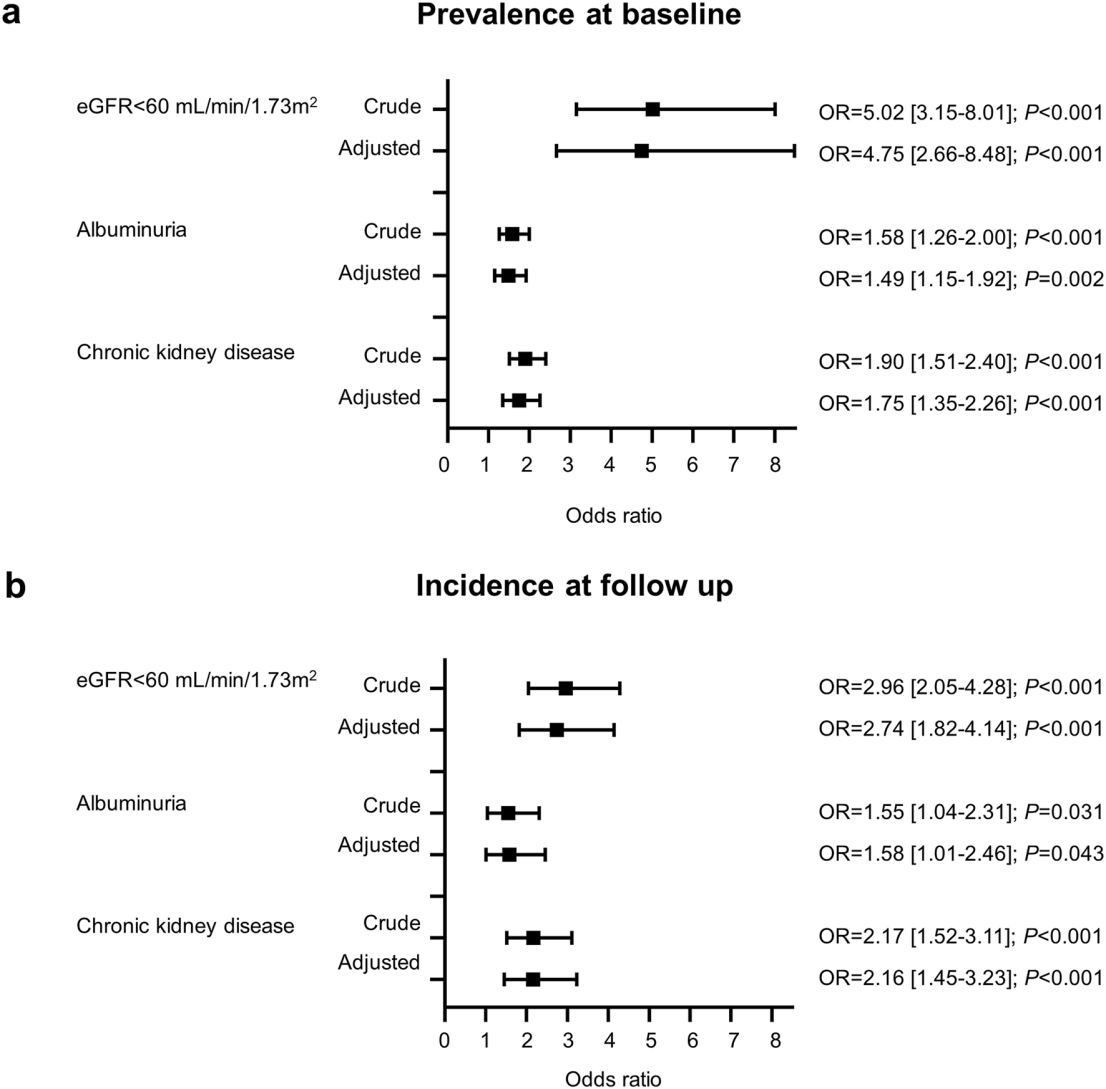
Associations between glypican-4 levels and (**a**) prevalence of eGFR < 60 mL/min/1.73 m^2^, albuminuria, and chronic kidney disease at baseline or (**b**) incidence of newly diagnosed eGFR < 60 mL/min/1.73 m^2^, albuminuria, and chronic kidney disease at follow up. Chronic kidney disease was attested in case of eGFR < 60 mL/min/1.73 m^2^ or albuminuria. Odds ratios with 95% confidence intervals [95%CI] per standard deviation of log-transformed GPC4 were obtained by univariable (crude) and multivariable logistic regression analyses adjusted for age, sex, body mass index, systolic and diastolic blood pressure, type 2 diabetes mellitus, history of smoking, C-reactive protein, and significant coronary artery disease.

Association of serum GPC4 with eGFR < 60 mL/min/1.73 m^2^, albuminuria, and CKD remained significant after correction for age, sex, BMI, T2DM, CAD, smoking, blood pressure, and C-reactive protein (CRP) in multivariable logistic regression analysis. Respective odds ratios with 95% confidence intervals for one standard deviation change of log-transformed GPC4 are illustrated in Fig. 2a.

Area under the receiver operating characteristic curve (ROC-AUC) analyses were used to compare the performance of GPC4 as a biomarker for kidney function with the new circulating biomarkers uromodulin and FGF23. GPC4 showed higher area under the curve (AUC) values for the diagnosis of eGFR < 60 mL/min/1.73 m^2^, albuminuria, and CKD at baseline as well as at follow up compared to uromodulin and FGF23 and, therefore, outperformed these markers for the diagnosis of baseline and future kidney function (Supplemental Table S2).

### Follow up

After a median follow up period of 3.4 [3.1–3.5] years, kidney function was reassessed in all patients. Serum GPC4 determined at baseline was significantly correlated with eGFR and ACR assessed at follow up (r = −0.406; *P* < 0.001 and r = 0.245; *P* < 0.001, respectively; adjusted results from analysis of covariance are shown in Supplemental Table S1).

eGFR < 60 mL/min/1.73 m^2^, albuminuria, and CKD were newly diagnosed in 50, 31, and 50, respectively, patients at follow up. GPC4 levels were significantly increased in patients with newly diagnosed eGFR < 60 mL/min/1.73 m2 at follow up compared to subjects with eGFR ≥ 60 mL/min/1.73 m2 at baseline as well as at follow up (6.97 [5.49–7.93] ng/mL versus 5.23 [4.42–6.28] ng/mL; *P* < 0.001). Similarly, patients with newly diagnosed albuminuria at follow up showed significant higher baseline GPC4 levels compared to patients with no albuminuria at baseline and at follow up (5.69 [4.86–7.09] ng/mL versus 5.27 [4.49–6.48] ng/mL; *P* = 0.042). Consequently, GPC4 levels were also significantly increased in patients with newly diagnosed CKD at follow up compared to patients with no CKD at baseline and at follow up (6.17 [5.12–7.70] ng/mL versus 5.13 [4.36–6.24] ng/mL; *P* < 0.001).

Logistic regression analyses showed that baseline GPC4 levels significantly and independently predicted the incidence of newly diagnosed eGFR < 60 mL/min/1.73 m^2^, albuminuria, and CKD during a 3.4-year period (Fig. 2b). GPC4 outperformed uromodulin, and FGF23 for the diagnosis of incident eGFR < 60 mL/min/1.73 m^2^, incident albuminuria, and incident CKD (Supplemental Table S3). ROC analysis further indicated an additional predictive value of GPC4 to a basic prediction model including baseline ACR, age, sex, BMI, blood pressure, T2DM, CRP, smoking, and the presence of CAD for incident CKD and incident eGFR < 60 mL/min/1.73 m^2^ (Table 3).

**Table 3:** Comparison of the area under the curve of prediction models for the incidence of eGFR < 60 mL/min/1.73 m2, albuminuria, and chronic kidney disease.

	Model	AUC (95% CI)	*P*-value	Z for DeLong’s test
New eGFR < 60 mL/min/1.73 m^2^	Basic	0.767 (0.699–0.835)	–	
	Basic + GPC4	0.831 (0.771–0.891)	0.004	−2.896
New Albuminuria	Basic	0.760 (0.673–0.847)	–	
	Basic + GPC4	0.782 (0.701–0.863)	0.211	−1.251
New CKD	Basic	0.678 (0.602–0.753)	–	
	Basic + GPC4	0.749 (0.679–0.818)	0.024	−2.253

Models were built as binary logistic regression models. The basic model comprises the albumin/creatinine concentration ratio, age, sex, BMI, blood pressure, type 2 diabetes mellitus, C-reactive protein, smoking, and presence of coronary artery disease. The basic model was compared with a model including variables of the basic model and additionally serum glypican-4. AUCs were compared according to DeLong’s test; respective *P-*values are given for the comparison with the basic model. AUC, area under the curve; CI, confidence interval; CKD, chronic kidney disease, GPC4, glypican-4.

## Discussion

In the present study, we showed significant associations between GPC4 and decreased eGFR, albuminuria, and as a consequence CKD in a cohort of coronary angiography patients. Previously, GPC4 has been associated with eGFR and urinary albumin excretion in a single, relatively small-sized study including 161 Korean patients with diabetes^21^. Our study does not only confirm these findings in a much larger cohort of patients but additionally shows that serum GPC4 predicts the future risk of decreased kidney function. In this regard, circulating GPC4 even outperforms other serum markers recently proposed for the detection of kidney disease such as uromodulin^5,7^ or FGF23^9,10^.

Our study is in line with previous findings demonstrating significant associations of serum GPC4 with BMI^13,17–19,23^ and systolic blood pressure^18^. The linkage between GPC4 and obesity-related traits may have contributed to the significant association between GPC4 and kidney function but does not fully explain it. Multivariable logistic regression analyses revealed that the association between GPC4 and kidney function remained robust when metabolic parameters and other conventional risk factors such as smoking or significant CAD were included in the model. Therefore, it can be assumed that GPC4 is linked with kidney function decline either as factor of kidney perfusion or as a product of unsatisfactory glycaemic or metabolic control beyond its association with a traditional kidney risk profile predisposing to CKD.

Increased GPC4 levels in patients with decreased kidney function may be a result of impaired glomerular filtration, responsible for elevated plasma levels of creatinine and urea as well. In fact, among the investigated parameters the strongest correlation was found between serum GPC4 und eGFR. However, unlike circulating creatinine and urea, serum GPC4 was strongly correlated with urinary albuminuria too. Albuminuria associated with progression of kidney disease is likely caused by the degradation of the glycocalyx, a hydrogel comprised of glycosoaminoglycans, glycoproteins and associated serum proteins that covers the luminal surface of the glomerular endothelium and that normally acts as a barrier against albumin filtration^24,25^. Degradation of the endothelial glycocalyx layer (“shedding”) occurs in inflammatory states and during ischemia and results in the release of measurable glycocalyx components into the circulation^26,27^. As GPC4 contributes to the formation of the glycocalyx, circulating GPC4, derived from endothelial glycocalyx shedding may, therefore, represent a surrogate marker of kidney dysfunction caused by damage to the glycocalyx. In fact, the glycocalyx shed markers syndecan-1 and hyaluronan have been previously associated with different CKD stages supporting this hypothesis^28,29^.

Since GPC4 is highly expressed in tubular epithelial cells in the adult kidney, it may also have a physiological function^30^. GPC4 can interact with the insulin receptor enhancing insulin signalling in preadipocytes and plays a role in adipocyte differentiation^13^. Insulin also performs distinct actions in kidney tissue that regulate metabolic and growth pathways as well as the kidney microcirculation. Consequently, resistance to the metabolic actions of insulin affects kidney structure and function^31^. It has been speculated that an increase of circulating GPC4 could represent a regulatory mechanism by which fat acts to counteract insulin resistance^13,32^. This hypothesis could be applicable for kidney tissue as well, assuming that increased GPC4 levels mirror elevated demand of insulin needed for maintenance of kidney function and kidney repair.

GPC4 is also involved in the control of the wingless/int-1 (Wnt) signalling pathway^33^, which triggers adaptive responses involved in kidney repair and regeneration, but may also promote disease progression, depending on the magnitude and duration of its activation^34–36^. The picture is even more complex, as membrane-bound GPC4 enhances Wnt signalling, but GPC4 secreted to the extracellular environment acts as competitive inhibitor of the Wnt signalling pathway^33^. Therefore, functional studies are needed to understand the biological role of circulating GPC4 in kidney disease.

Our study has several strengths and limitations. By design, our study population was composed of angiography coronary patients of European ancestry; our results therefore are not necessarily applicable to other ethnicities or the general population. However, the high-risk patient population of coronary angiography patients we chose to investigate is of particular clinical interest. The prospective part of our study represents the first observation linking GPC4 to the future decline of kidney function. However, GPC4 levels have been associated with various obesity-related traits^13,17–21,23^ and, therefore, may not be specific to the malfunction of a particular organ. In addition, despite the statement of the manufacturer, that the used ELISA shows high specificity to human GPC4, any assay cross- reactivity to GPC4 analogues cannot be fully excluded. Therefore, the specificity of the GPC4 ELISA needs to be confirmed before it could be used as a predictive biomarker in the future. Importantly, due to the observational design of the present study, the pathophysiological mechanisms of the association between circulating GPC4 and impaired kidney function remain speculative and need to be investigated in further studies.

In summary, our study shows that serum GPC4 is strongly associated with kidney function-related traits and for the first time predicts the development of CKD. The value of GPC4 as a new biomarker for kidney malfunction and kidney disease progression has to be further evaluated in subsequent studies.

## Methods

### Study population

From September 2005 through April 2008, we enrolled 1048 consecutive Caucasian patients who were referred to elective coronary angiography for the evaluation of established or suspected stable CAD at the Academic Teaching Hospital Feldkirch, Austria. Out of these, 456 subjects had available serum samples for GPC4 analysis and data on kidney function at baseline as well as at follow up. Patients with acute kidney injury were not included. Coronary angiography was performed with the Judkin's technique and coronary artery stenoses with stenotic narrowing ≥ 50% were defined as significant CAD^37^. Hypertension was defined according to the Seventh Report of the Joint National Committee on Prevention, Detection, Evaluation, and Treatment of High Blood Pressure^38^. T2DM was diagnosed according to American Diabetes Association criteria^39^. The study has been approved by the Ethics Committee of the University of Innsbruck, Austria, and written informed consent was given by all participants before taking part in the study. All methods were performed in accordance with the relevant guidelines and regulations.

### Laboratory measurements

Venous blood samples were collected after an overnight fast of 12 h before angiography was performed. Routine blood variables were determined in fresh serum samples by standard laboratory methods at the Medical Central Laboratories, Feldkirch, Austria. Serum samples were stored at −80 °C until used for analysis of FGF23, uromodulin, and GPC4. FGF23 measurements were performed at Immundiagnostik AG (Bensheim, Germany) and serum uromodulin was detected as previously described^7^. Serum GPC4 levels were determined using the same batch of a commercial enzyme-linked immunosorbent assay (ELISA) kit (Cloude-Clone, Houston, Texas; article number: SEA998Hu) following the manual of the manufacturer. The kit had a detection range of 0.031–2.000 ng/mL; the intra-assay coefficient of variation (CV) was below 10% and the inter-assay CV was below 12%. Serum samples were diluted 1:4 using phosphate buffered saline as a dilution buffer prior to analysis.

### Parameters of kidney function

GFR was estimated (eGFR) using the ‘Chronic Kidney Disease Epidemiology Collaboration’ (CKD-EPI) serum creatinine equation. GFR categories were defined as follows: G1: eGFR ≥ 90 mL/min/1.73 m^2^; G2: eGFR = 60–89 mL/min/1.73 m^2^; G3a: eGFR = 45–59 mL/min/1.73 m^2^; G3b: eGFR = 30–44 mL/min/1.73 m^2^; G4: eGFR = 15–29 mL/min/1.73 m^2^. Urinary albumin excretion was expressed as ACR in a random morning urine specimen.

Urinary albumin concentration was determined by immunoturbidometry (Tina-quant Albumin Gen.2 Assay, Roche Diagnostics, Vienna, Austria). Both serum and urinary creatinine concentrations were measured with a modified Jaffé method (Creatinine Jaffé Gen.2 Assay, Roche Diagnostics). ACR levels lower than 30 mg/g (category A1) were defined as normal and albuminuria was diagnosed as an ACR of 30 mg/g or greater. Moderately increased albuminuria (category A2; formerly microalbuminuria) was defined as an ACR of 30 to 300 mg/g and severely increased albuminuria (category A3; formerly macroalbuminuria) as an ACR of 300 mg/g or greater. CKD was diagnosed in case of eGFR ˂ 60 mL/min/1.73 m^2^ or albuminuria. Due to the observational design of the study, our definition of CKD was based on single measurements of kidney function. Prognosis of CKD was classified into four risk-categories according to the classification system proposed by the 2012 KDIGO recommendations^4^, which were based on GFR categories (G1-G5) and ACR categories (A1-A3) and were as follows: Low risk: G1 and A1 or G2 and A1; moderately increased risk: G1 and A2, G2 and A1, G2 and A2 or G3a and A1; high risk: G1 and A3, G2 and A3, G3a and A2 or G3b and A1; very high risk: G3a and A3, G3b and A2, G3b and A3, G4 or G5.

### Prospective study

After a median period of 3.4 years, patients were re-invited to undergo a follow-up investigation and kidney function was reassessed.

### Statistical analyses

Differences in baseline characteristics according to GPC4 quartiles were tested for statistical significance with Chi-squared tests for trend for categorical and Jonckheere Terpstra tests for continuous variables, respectively. Continuous variables are given as median and interquartile range (defined as the range from the 25th percentile to the 75th percentile. Normal distribution was assessed using Kolmogorov–Smirnov and Shapiro–Wilk test, respectively, concluding that GPC4 values were not normally distributed. Correlation analyses were performed calculating non-parametric Spearman rank correlation coefficients. In addition, analysis of covariance models were built using a general linear model approach. Non-normally distributed variables were log-transformed by the base of 10 before they were entered into parametric models. Statistically significant differences between GPC4 levels and categorical variables were determined by the Mann–Whitney U test and the Jonckheere Terpstra test, respectively. Odds ratios and 95% confidence intervals [95%CI] were obtained from univariable and multivariable logistic regression analyses adjusting for age, sex, BMI, blood pressure, T2DM, smoking, CRP, and the presence of CAD. Z-transformation was applied before logistic regression analysis. All data were analysed according to complete-case analysis due to neglible percentage of missing data. Additionally, ROC-AUC analyses were applied to compare the performance of GPC4 as a diagnostic biomarker with other kidney markers. To examine the potential utility of GPC4 as a predictive biomarker, binary logistic regression models were fitted with eGFR < 60 mL/min per 1.73 m^2^, albuminuria, and CKD, respectively, as the dependent variable. A basic model comprising ACR, age, sex, blood pressure, BMI, T2DM, CRP, smoking history, and the presence of significant CAD as independent variables was compared with a second model including GPC4 as an additional marker. Receiver operating characteristic and the respective AUC were calculated. The statistical significance of the difference between AUCs was tested with the method of DeLong^40^. All statistical analyses were performed with SPSS 27.0 (SPSS, Inc., Chicago, IL) software.

## Data availability

The datasets generated and/or analysed during the current study are available in the Mendeley Data repository, ‘Data on the association of serum glypican-4 with prevalent and future kidney function’ (https://doi.org/10.17632/nx94z23388.1; https://data.mendeley.com/datasets/nx94z23388/1).

## Supplementary Information

Supplementary Information.
