# Supplementary material for: Evaluation of the association of serum glypican-4 with prevalent and future kidney function
Source: Sci Rep. 2022 Jun 17;12:10168. doi: 10.1038/s41598-022-14306-7 (PMC9206029; doi:10.1038/s41598-022-14306-7)
Supplement: Supplementary file 1 — Supplementary Information. [file 41598_2022_14306_MOESM1_ESM.docx]

**Supplemental Material**

**Evaluation of the association of serum glypican-4 with prevalent and future kidney function**

**Axel Muendlein^a^, Eva Maria Brandtner^a^, Andreas Leiherer^a,b,c^, Kathrin Geiger^a,b^, Christine Heinzle^a,b^, Stella Gaenger^a^, Peter Fraunberger^b^, Dominik Haider^d^, Christoph H. Saely^a,c^, Heinz Drexel^a,c,d,e^**

^a^Vorarlberg Institute for Vascular Investigation and Treatment (VIVIT), Feldkirch, Austria; ^b^Medical Central Laboratories, Feldkirch, Austria; ^c^Private University of the Principality of Liechtenstein, Triesen, Liechtenstein; ^d^Department of Medicine, Academic Teaching Hospital Bregenz, Bregenz, Austria; ^e^Drexel University College of Medicine, Philadelphia, PA, USA

**Supplemental Table S1: Analyses of covariance for the association of serum glypican-4 with estimated glomerular filtration rate and the albumin/creatinine concentration ratio at baseline and follow-up**

|  |  | Baseline | |  | Follow Up | |
| --- | --- | --- | --- | --- | --- | --- |
|  |  | F | *P* |  | F | *P* |
| eGFR | Unadjusted | 132.7 | < 0.001 |  | 125.4 | < 0.001 |
|  | Model 1 | 85.6 | < 0.001 |  | 78.9 | < 0.001 |
|  | Model 2 | 79.0 | < 0.001 |  | 77.5 | < 0.001 |
|  |  |  |  |  |  |  |
| ACR | Unadjusted | 24.5 | < 0.001 |  | 31.3 | < 0.001 |
|  | Model 1 | 13.0 | < 0.001 |  | 19.5 | < 0.001 |
|  | Model 2 | 13.7 | < 0.001 |  | 19.5 | < 0.001 |

Model 1: adjusted for age, sex, and body mass index; Model 2: adjusted for age, sex, body mass index, systolic and diastolic blood pressure, type 2 diabetes mellitus, history of smoking, C-reactive protein, and coronary artery disease. Continuous variables were not normally distributed and log-transformed by the base of 10 before being factored into analyses of covariance. eGFR, estimated glomerular filtration rate; ACR, albumin/creatinine concentration ratio.

**Supplemental Table S2: Comparison of the performance of glypican-4 as a biomarker for baseline kidney function with other serum markers**

|  |  | Prevalence of eGFR< 60 mL/min/1.73 m^2^ | |  | Prevalence of albuminuria | |  | Prevalence of CKD | |
| --- | --- | --- | --- | --- | --- | --- | --- | --- | --- |
|  |  | AUC [95%CI] | *P*-value |  | AUC [95%CI] | *P*-value |  | AUC [95%CI] | *P*-value |
| Glypican-4 |  | 0.860 [0.793-0.028] | <0.001 |  | 0.630 [0.567-0.694] | <0.001 |  | 0.673 [0.613-0.732] | <0.001 |
| Uromodulin |  | 0.813 [0.733-0.893] | <0.001 |  | 0.561 [0.482-0.640] | 0.131 |  | 0.601 [0.526-0.676] | 0.008 |
| FGF23 |  | 0.801 [0.724-0.877] | <0.001 |  | 0.575 [0.504-0.646] | 0.037 |  | 0.604 [0.536-0.672] | 0.003 |

Results from area under the receiver operating characteristic curve (ROC-AUC) analyses. The higher the AUC value, the higher the predictive value of the biomarker. All biomarkers were measured in serum or plasma samples. Chronic kidney disease was diagnosed in case of eGFR˂60 ml/min/1.73 m^2^ or albuminuria. CKD, chronic kidney disease; AUC, area under the curve; CI, confidence interval; FGF23, fibroblast growth factor 23.

**Supplemental Table S3: Comparison of the performance of glypican-4 as a biomarker for kidney function at follow up with other serum markers**

|  |  | Incidence of eGFR< 60 mL/min/1.73 m^2^ | |  | Incidence of albuminuria | |  | Incidence of CKD | |
| --- | --- | --- | --- | --- | --- | --- | --- | --- | --- |
|  |  | AUC [95%CI] | *P*-value |  | AUC [95%CI] | AUC [95%CI] |  | AUC [95%CI] | *P*-value |
| Glypican-4 |  | 0.763 [0.696-0.830] | <0.001 |  | 0.610 [0.512-0.708] | 0.028 |  | 0.681 [0.603-0.759] | <0.001 |
| Uromodulin |  | 0.612 [0.511-0.713] | 0.030 |  | 0.434 [0.303-0.564] | 0.317 |  | 0.507 [0.401-0.613] | 0.899 |
| FGF23 |  | 0.605 [0.520-0.690] | 0.016 |  | 0.589 [0.482-0.696] | 0.102 |  | 0.550 [0.464-0.637] | 0.255 |

Results from area under the receiver operating characteristic curve (ROC-AUC) analyses. The higher the AUC value, the higher the predictive value of the biomarker. All biomarkers were measured in serum or plasma samples. Chronic kidney disease was diagnosed in case of eGFR˂60 ml/min/1.73 m^2^ or albuminuria. CKD, chronic kidney disease; AUC, area under the curve; CI, confidence interval; FGF23, fibroblast growth factor 23.
